# Experimentally Induced Language Modes and Regular Code-Switching Habits Boost Bilinguals’ Executive Performance: Evidence From a Within-Subject Paradigm

**DOI:** 10.3389/fpsyg.2020.542326

**Published:** 2020-11-05

**Authors:** Julia Hofweber, Theodoros Marinis, Jeanine Treffers-Daller

**Affiliations:** ^1^Department of Psychology and Human Development, Institute of Education, University College London, London, United Kingdom; ^2^School of Psychology and Clinical Language Sciences, University of Reading, Reading, United Kingdom; ^3^Department of Linguistics, University of Konstanz, Konstanz, Germany; ^4^Department of English Language and Applied Linguistics, University of Reading, Reading, United Kingdom

**Keywords:** code-switching, language modes, multilingualism, executive functions, cognition

## Abstract

Bilingualism may modulate executive functions (EFs), but the mechanisms underlying this phenomenon are poorly understood. In this study, we investigated two potential sources of variability in bilinguals’ EF performance: (1) interactional contexts and code-switching, and (2) dominance profiles. Previous research on code-switching often relied on self-reports of regular code-switching habits. In this study, we investigated the effects of experimentally induced language modes (single language versus code-switching modes) on bilinguals’ EF performance. Crucially, in the bilingual conditions, we differentiated between different types of intra-sentential code-switching (Insertion, Alternation, and Dense code-switching). Moreover, we investigated the interaction of the effects of temporary language modes with bilinguals’ sociolinguistic code-switching habits. All our participants were L1-dominant German–English bilinguals (*N* = 29) immersed in an L2 context. We assessed the effects of dominance by correlating individual bilinguals’ L1-dominance with their EF performance. In addition, we investigated whether language modes activate different EF patterns in bilinguals, as opposed to monolinguals, i.e., individuals who have no additional language to suppress. Based on models of bilingual language processing, we predicted our bilinguals to display the best EF performance in L2 single language contexts, as these require them to activate inhibitory schemata to suppress their dominant L1. Indeed, bilinguals performed better in the single language than in the code-switching conditions. The results also suggested that bilinguals activated more inhibitory control compared to monolinguals, supporting the notion that bilingual processing involves inhibition. The task conditions inducing different code-switching modes differed only in terms of the predictors explaining EF performance in the regression. We observed negative correlations between the frequency of engaging in a given type of code-switching and performance in language modes inducing non-corresponding control modes. The results suggested that Dense code-switching draws upon proactive control modes that differ from the reactive control involved in Alternation. Importantly, bilinguals’ dominance profiles played a crucial role in explaining EF performance. The more balanced individuals in our overall L1-dominant sample displayed better EF performance in the bilingual conditions, suggesting that more balanced bilingualism trains the control modes involved in code-switching. This highlights the importance of assessing bilinguals’ sociolinguistic profiles in bilingualism research.

## Introduction

Some studies suggest that bilingualism modulates individuals’ executive functions (EFs) because the demands of managing more than one language challenge and train cognitive control (Bialystok, 2017). However, results in the field of bilingualism and executive functions have been inconsistent, with some large-scale studies finding no interaction between bilingual processing and EFs (Paap and Greenberg, 2013; Duñabeitia and Carreiras, 2015; Lehtonen et al., 2018). To achieve their large sample sizes, many of these studies have combined different types of bilinguals into the same group. Bilingualism, however, is a multi-faceted phenomenon that comprises many component parts and sub-groups of different types of bilinguals (Luk and Bialystok, 2013). Combining different types of bilinguals and treating them as a single group means ignoring the large individual variability that is caused by differences in the bilinguals’ language history and linguistic profile, introduces noise in the data, and increases the likelihood of a Type II error. Hence, it is not only group size that matters, but also a thorough understanding of participants’ language history and language usage patterns (Khodos and Moskovsky, 2020). To avoid this issue, the present study focused on a specific type of bilinguals and mapped the language history and language usage profiles of each individual participant.

The amount of cross-linguistic competition and resulting inhibitory effort involved in bilingual processing depends on a multiplicity of factors, such as the typological distance of languages, the interactional contexts in which they are used, as well as bilinguals’ relative dominance and proficiency in the two languages (Duñabeitia and Carreiras, 2015; Bak, 2016). In this study, we focused on two such aspects, which have been put forward as potential sources of variability in bilinguals’ EF performance: (1) the interactional context in which bilinguals use their languages, that is either a single-language or a bilingual context, and (2) bilinguals’ language dominance profiles. Importantly, we carefully assessed bilinguals’ language history and language usage profiles. This allowed us to tailor our predictions and conclusions specifically to the linguistic profile of our bilingual group, as well as to assess individual variability within the sample. Bilinguals’ language combination (L1-German and L2-English) was kept constant to avoid variability due to differences in typological distance which are known to affect bilingual practices and processing (Muysken, 2000). Moreover, all bilinguals in this study were L1-dominant sequential bilinguals immersed in an L2-context (United Kingdom). At the same time, they displayed individual variability in their level of L1-dominance.

The main aim of this study was to investigate the extent to which experimentally-induced language modes affect bilinguals’ executive functioning. We assessed the impact of different interactional settings on bilinguals’ EFs by manipulating the relevant activation levels of languages within the experimental setting itself. This allowed us to observe the effects of single versus mixed language modes on EFs within the same subject. Thus, we avoided the potential confounds associated with comparing different groups of bilinguals (Soveri et al., 2011). In addition, we also investigated the interaction of these temporary effects with bilinguals’ regular code-switching habits as measured in a frequency judgment task. It should be noted nonetheless that some group distinctions remain necessary. Different linguistic phenotypes should not be conflated with traditional individual difference analyses (Green et al., 2006; Beatty-Martínez and Dussias, 2017; Hofweber et al., 2019; Beatty-Martínez et al., 2020). In this study, we conducted an additional group comparison between bilinguals and a group of participants whose sociolinguistic background can be characterized as “functionally monolingual”. To assess whether any potential within-subject variability amongst the bilinguals may arise from their need to suppress another language, we compared bilinguals’ EF performance in the single-language condition to that of a monolingual baseline group who had no additional language to suppress.

### Processing Models Describing the Impact of Different Language Modes on EFs

Bilinguals operate in different language modes, ranging from single language modes to code-switching contexts in which languages are mixed to differing degrees (Grosjean, 2001). This requires them to flexibly adapt the control modes they operate in to different interactional contexts and interlocutors, depending on a range of factors (Kroll et al., 2006). According to existing processing models of code-switching, such as the Adaptive Control Hypothesis ACH (Green and Abutalebi, 2013) and the Control Processing Model of Code-switching CPM (Green and Wei, 2014), different interactional contexts trigger different types of control modes. The ACH differentiates between single language contexts, dual language contexts (inter-sentential code-switching) and dense code-switching (intra-sentential code-switching). Crucially, the ACH posits that EFs are challenged most in so-called “dual language contexts,” in which bilinguals are switching languages between sentences, for instance to accommodate different interlocutors. Intra-sentential code-switching is predicted to engage less EFs because none of the involved languages are suppressed. This hypothesis is supported by Sanchez-Azanza et al. (2020), who found language switching to have positive effects on EFs amongst Spanish-Catalan bilinguals who function in a dual language context. Although the Sanchez-Azanza et al. (2020) study provides insights into the relationship between dual context language switching and EFs, it did not investigate the relative impact of inter- and intra-sentential code-switching on EFs. When comparing the effects of intra-sentential and inter-sentential effects on EFs, Hartanto and Yang (2020) found both intra- and inter-sentential code-switching to modulate EFs. This finding was in contrast to the ACH and their own predictions, which had been based on the ACH. Moreover, a study by Hofweber et al. (2016) found dense forms of code-switching to positively predict EFs. Clearly the different outcomes make it necessary to further test the impact of different interactional contexts on EFs among a wider range of bilinguals.

Sociolinguistic corpora suggest that much bilingual code-switching is in fact intra-sentential, so in this study we focused on investigating intra-sentential code-switching, deriving our predictions from the Control Processing Model of Code-switching CPM (Green and Wei, 2014), which makes testable hypotheses about the involvement of EFs in intra-sentential code-switching and single language contexts. The CPM contrasts (a) diglossic settings, in which bilinguals operate only in single-language contexts, i.e., using language A at home and language B at work, with (b) contexts in which bilinguals regularly mix languages. Bilinguals operate in a single-language mode when they converse with speakers they share only one language with, restricting themselves to the shared language. The target language in such single-language modes is selected by suppressing the non-target language, which involves high levels of inhibitory control (Green, 1998; Green and Wei, 2014). According to the CPM, single-language contexts therefore trigger competitive control modes involving high levels of inhibition to suppress the non-target language, whilst inhibition operates to a lesser extent in code-switching contexts, during which inhibition is lifted.

Based on the CPM, we predicted bilinguals to display greater levels of inhibitory activation in single-language modes than in bilingual modes. Moreover, we predicted that this effect would be unique to bilinguals, which should translate into performance differences in comparison with the monolingual baseline group. If bilinguals recruit inhibition for the purpose of language control, then they should display greater activation of inhibition than monolinguals, who have no additional language to suppress. Different sociolinguistic contexts favor different prevalent code-switching patterns and resulting pre-dominant interactional modes (Muysken, 2000). Diglossia is typical of contexts in which clear lines are drawn between different lingua-cultural contexts, often for socio-political reasons. However, many bilingual contexts also involve situations in which bilinguals use both languages within the same conversation, or indeed within the same sentence. This means that in the presence of other bilinguals sharing the same language combination, bilinguals often converse in bilingual modes (Grosjean, 2001). In bilingual modes, they draw upon their multilingual competence, mixing languages to optimally get their message across. This behavior is referred to as “code-switching” (Bhatt and Bolonyai, 2011). In this study, we focused on intra-sentential code-switching. According to the CPM, intra-sentential code-switching triggers co-operative control modes, which involve reduced levels of inhibition as the languages remain co-activated. The level of linguistic co-activation and resulting inhibitory involvement depends on the nature of the code-switches. The more a given form of code-switching keeps the languages separate, the greater are the levels of inhibition involved (Treffers-Daller, 2009). This suggests that it is not sufficient to talk about a singular “bilingual mode,” but that there are at least three different code-switching types, which have been shown to differ in EF involvement (Green and Wei, 2014; Hofweber et al., 2016, 2020). The exact control modes activated in the bilingual mode will depend on the nature of the code-switching bilinguals engage in (Treffers-Daller, 2009; Green and Wei, 2014; Hofweber et al., 2020).

The present study differentiated between three different code-switching patterns, described by Muysken (2000): Alternation, Insertion and Dense code-switching (Congruent Lexicalisation). Dense code-switching involves the activation of both languages at both the grammatical and lexical levels, as in (1), where the expression *Wir haben friends gemacht* is not a usual German expression but a calque (literal translation) from English *We have made friends*. Note that the word order has been adapted to German because *friends* appears before the verb instead of after it, although the PP *mit’m shopowner* would also normally be preverbal in standard German. Thus, the word order is a compromise between English and German and words from either language are combined in the shared grammatical structure. Alternation involves switching between longer and fairly independent stretches of language, as in (2). The term Insertion describes the import of lexical items from an embedded language into a matrix language, which consistently provides the grammatical frame of the bilingual’s utterances, as in (3), where English *degree* is inserted into a structure in which German is clearly the matrix language.

(1) Wir haben *friends* gemacht mit’m *shop owner*.

We have friends made with th’ shop owner.

“We have made friends with th’ shop owner.”

(2) Ich gehe erst heim *to drop some stuff off*.

“I am going home first *to drop some stuff off.”*

(3) Meinen *degree* habe ich in England gemacht.

“I did my degree in England”.

The CPM offers a purely quantitative account of inhibitory involvement in code-switching, suggesting that the greatest levels of inhibition are involved in Alternation, followed by Insertion, followed by Dense code-switching. However, existing studies on code-switching and EFs highlight the importance of differentiating between qualitatively different sub-components of EFs (Hofweber et al., 2016, 2020; Hartanto and Yang, 2019; Gullifer and Titone, 2020a; Khodos and Moskovsky, 2020). To describe the EF processes involved in the different language modes, we therefore followed Green and Abutalebi (2013) and Hofweber et al. (2020) in drawing upon Braver’s (2012) dual control framework. Braver (2012) describes two different control modes. The term control mode refers to the high-level EF processes that co-ordinate the de- and re-activation of inhibition to manage competing task schemata (Botvinick et al., 2001). Braver’s framework differentiates between “reactive” and “proactive” control modes, which represent the extreme ends of a continuum. Individuals shift along this continuum “according to whether interference can be anticipated or not” (Braver, 2012, p. 107). When a situation requires infrequent task-switching, individuals operate in a reactive control mode, in which non-target schemata are globally inhibited. This infrequent activation of inhibitory control is effortful, so when a situation requires frequent task-switching, it becomes more efficient to transition to proactive control modes, in which task-schemata remain latently co-activated, which challenges monitoring. Crucially, Braver claims that “changes in situational factors will affect the weighting of proactive versus reactive strategies” (Braver, 2012, p. 107). Support for this analysis can be obtained from Gullifer and Titone (2020b) who looked at the relationship between bilinguals’ use of proactive control strategies on different versions of the Flanker task and the degree of compartmentalization across contexts in their everyday speech. The authors found that high entropy bilinguals (that is those who used different languages within one context) were more likely to use proactive control strategies than low entropy bilinguals (who separate languages strictly by context).^1^

**FIGURE 1:**
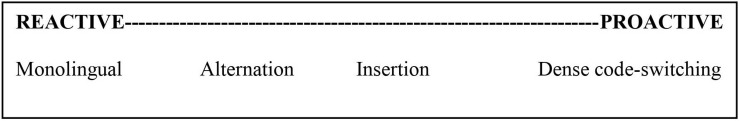
Reactive-proactive control continuum.

Following this logic, Hofweber et al. (2020) refer to the dual control framework to explain changes in cognitive control demands posed by different interactional contexts and language modes. Single-language modes map onto reactive control processes because bilinguals need to globally suppress the non-target language, which comes at a high inhibitory cost. Bilingual code-switching modes, on the other hand, trigger more proactive control modes because interlocutors manage linguistic co-activation. This comes at a high monitoring cost. Thus, the different bilingual modes are predicted to differ not only in terms of the quantity but also in terms of the quality of inhibitory control and monitoring involvement. Dense code-switching should trigger the most proactive control mode because linguistic co-activation needs to be monitored. At the other end of the continuum, Alternation involves switching between longer and structurally more independent stretches of language, which should trigger relatively more reactive control modes involving inhibitory control to transition from one language to another. Insertion is predicted to involve mostly reactive control, as the embedded language remains largely suppressed and cross-linguistic influence is limited to the lexical level. Indeed, the CPM suggests that Insertion and Alternation draw upon similar control mechanisms, labeling these as “Coupled Control modes.” In line with this reasoning, we assume that Alternation and Insertion both involve reactive control modes, although the precise nature of the control modes may differ along the reactive-proactive control continuum. Single-language modes should draw upon the most reactive forms of inhibition because the non-target language remains suppressed for prolonged periods of time. To summarize, we predicted the different language modes to trigger transitions along the reactive-proactive control continuum, as described in Figure 1.

### Evidence From Experimental Paradigms Inducing Different Language Modes

In this study, we aimed to experimentally elicit these different language modes in our bilinguals and assess their interaction with EF performance. Our experimental paradigm is based on a study by Wu and Thierry (2013), which showed that experimentally induced language modes can temporarily alter executive functioning. Using a novel experimental paradigm, Welsh–English bilinguals performed a flanker task measuring inhibitory control in different language modes. In the single-language conditions, the flanker trials were interspersed by only English or Welsh words, respectively. In the bilingual condition, English and Welsh words alternated, inducing a bilingual mode. Inhibitory performance was assessed by comparing performance in flanker trials requiring inhibition to those that do not require inhibition, using both behavioral and electrophysiological measures (P300 reflecting inhibitory effort). Although RTs did not differ across the three task conditions, participants showed enhanced inhibitory performance (reduced error rates and P300 amplitudes) in the bilingual compared to the single-language condition.

The authors explained this finding by drawing upon literature that suggests that when a specific tasks activates EFs, this effect can have positive transfer effects on other simultaneously performed tasks (Botvinick et al., 1999). Thus, they deduced that the bilingual mode activated participants’ EFs, which positively affected performance at the non-verbal inhibitory task element. This is in line with neuroimaging data revealing overlaps in brain regions activated during non-verbal conflict resolution and tasks challenging language control (Green and Abutalebi, 2007). These cross-fertilization effects between verbal and non-verbal executive functioning demonstrate the responsiveness of EF networks to participants’ current language modes, which is indicative of functional plasticity and *fast-modulation* effects of language modes on EFs.

Bilinguals’ better inhibitory performance in the bilingual mode in Wu and Thierry’s (2013) study is not entirely in line with the CPM, which would suggest greater levels of inhibitory activation in the single-language condition involving the suppression of the non-target language. It is possible that the effects of fast-modulation depend on the interactional contexts prevalent in bilinguals’ sociolinguistic context. In fact, in reference to the Wu and Thierry (2013) study, the CPM specifically predicts “a cross-over interaction with the effects of local verbal context on conflict in the flanker task contingent on interactional contexts of the speakers” (Green and Wei, 2014, p. 506). Studies based on similar experimental paradigms have since shown that the effects of fast-modulation vary as a function of bilinguals’ sociolinguistic habits and language dominance profiles. A study by Bosma and Pablos (2020) thus shows that the reading of code-switches engages EFs, and that inhibitory control is most involved when the suppression of a dominant L1 is required. This is in line with several studies that have found that inhibitory costs are asymmetric, i.e., greater when suppressing the L1 than the L2 (Meuter and Allport, 1999; Costa and Santesteban, 2004; Filippi et al., 2014). It has therefore been argued that dominant bilingualism trains certain forms of inhibitory control to a greater extent than balanced bilingualism (Goral et al., 2015). The English-Welsh bilinguals in Wu and Thierry’s (2013) study were balanced bilinguals who acquired both languages simultaneously from an early age and used both languages equally frequently. This may explain why the single-language condition did not trigger heightened levels of inhibition in these balanced bilinguals. In the case of L1-dominant bilinguals, one could hypothesize better performance in the single-language condition, especially in conditions requiring the suppression of the dominant L1.

For L1-dominant bilinguals, the impact of experimentally induced language modes on EFs has so far only been investigated in one study by Jiao et al. (2020). They administered flanker trials interspersed with picture naming tasks, comparing a single language condition to a mixed language condition. Although no effects were observed behaviorally, the ERP results suggested a boost of EFs in the mixed language mode. These findings could be due to bilinguals’ language background and sociolinguistic practices. The bilinguals in the Jiao et al. (2020) study were non-immersed L2 users of English who were strongly dominant in their L1 Chinese. Their use of the L2 English was infrequent and limited to formal settings, such as lectures. It is therefore unlikely that these bilinguals regularly engaged in code-switching, which could explain why they found the mixed language mode more challenging than the single language mode, resulting in an EF activation. This sets them apart from the L1-dominant bilinguals in this study who were fully functional bilinguals immersed in an L2 context, and who were regular users of code-switching. The long-term immersed bilinguals in this study were expected to find bilingual modes less effortful, and activate greatest levels of inhibitory control when suppressing their dominant L1.

A limitation of the Wu and Thierry (2013) and the Jiao et al. (2020) studies is the vagueness of the term “bilingual mode.” Language switching is only investigated at the word-level, when in reality the code-switching discussed by the CPM also happens at the sentence level, and involves intricate grammatical consolidation processes (Muysken, 2000). It could be argued that the studies by Wu and Thierry (2013), and Jiao et al. (2020) induced inter-sentential switching modes as bilinguals were switching between languages but not within the same sentence. The predictions of the CPM therefore only apply to the Wu and Thierry (2013) and the Jiao et al. (2020) studies to a limited extent. A study by Adler et al. (2020) explored the effects of sentential code-switching on EFs amongst a group of Spanish–English bilinguals, using ecologically valid sentential code-switches. They interspersed flanker trials alternatingly with sentences containing code-switches and single-language sentences. The sentences were presented using a self-paced reading paradigm. The bilinguals performed better in flanker trials immediately preceded by a code-switch than in flanker trials preceded by single-language sentences, suggesting that the reading of code-switching activated cognitive control processes.

Although the Adler et al. (2020) study provides interesting insights into the differences between the EF involvement in code-switching versus single-language contexts, it did not systematically control for different types of intra-sentential code-switching. Moreover, the Adler et al. (2020) study may also have induced an inter-sentential code-switching mode, in which bilinguals were continuously switching between single and mixed language modes. However, as explained in the sections above, real-life intra-sentential code-switching displays different levels of grammatical integration (Muysken, 2000; Clyne, 2003), and the amount of grammatical consolidation required in code-switching impacts the EFs involved (Green and Wei, 2014; Hofweber et al., 2016). Hence, the frequency and density of code-switching has been argued to trigger different control modes, i.e., proactive versus reactive control modes (Hofweber et al., 2020). There is therefore a need to differentiate not only between monolingual and bilingual modes, but also between different types of intra-sentential code-switching. In this study, we presented different types of intra-sentential code-switching in a blocked design, to allow bilinguals to “get into” a certain code-switching mode by being exposed to 96 trials in a row of each type of code-switching.

It is important to distinguish between experimentally-induced language contexts in the same bilinguals (Wu and Thierry, 2013; Blanco-Elorrieta et al., 2018; Adler et al., 2020; Jiao et al., 2020), and examining different bilinguals as a function of their interactional experience (Hartanto and Yang, 2016; Hofweber et al., 2016; Beatty-Martínez and Dussias, 2017; Gullifer et al., 2018; Kroll et al., 2018; Beatty-Martínez et al., 2020). In this study, we aimed to combine insights from both approaches by investigating how bilinguals’ regular code-switching habits interacted with their EF performance in the different experimentally induced language modes. While shifting between language modes is a fundamental ability that bilinguals of all language backgrounds are equipped with, how individuals adapt to resolve a control dilemma will likely vary as a function of language experience (Beatty-Martínez and Dussias, 2017; Beatty-Martínez et al., 2020).

In this context, it is important to note that the prevalence of different intra-sentential code-switching patterns and language modes differs as a function of bilinguals’ sociolinguistic context (Muysken, 2000). Sociolinguistic patterns are community specific (Muysken, 2000) and code-switching speech practices differ across communities and individuals even within the same language pair (Beatty-Martínez et al., 2018; Beatty-Martínez and Dussias, 2019). Whilst Insertion and Alternation are common in most bilingual contexts, Dense code-switching is limited to established bilingual communities (Hofweber et al., 2016). Existing studies on experimentally induced language modes have not taken into account bilinguals’ sociolinguistic background (Wu and Thierry, 2013; Jiao et al., 2020). Although Wu and Thierry (2013) did not explicitly control for sociolinguistic usage patterns, Green and Wei (2014) make predictions as to how regular code-switching habits may have affected the results in the case of the Wu and Thierry’s (2013) study. According to the ACH and the CPM, Alternation and Insertion involve greater levels of inhibition than Dense code-switching. Hence, Green and Wei (2014) hypothesize that, in order for bilingual modes to trigger heightened levels of inhibition, the Welsh–English bilinguals must have regularly engaged in Insertion or Alternation, because these are the types of code-switching that involve high levels of inhibition. Indeed, sociolinguistic analyses of Welsh–English code-switching corpora reveal language mixing to be predominantly Insertional in nature in this speech community (Deuchar et al., 2008). If the bilinguals had been frequent Dense code-switchers, the mixed language mode would have triggered low levels of inhibition. In our study, we explicitly measured bilinguals’ regular code-switching habits using a frequency judgment task. Although we did not compare different sociolinguistic contexts, we therefore acknowledged the importance of regular code-switching practices by assessing the role of individual variability in code-switching usage.

### The Role of Bilinguals’ Language Dominance and Immersion Profile

In addition to investigating the impact of different language modes on EFs, the present study aimed to shed light on the interaction between language dominance and EFs. Some studies suggest that EF enhancements are reserved to balanced bilinguals (Luk et al., 2011; Yow and Li, 2015). Indeed, the brain regions involved in conflict-monitoring have been shown to differ in early balanced compared to late sequential bilinguals (Mohades et al., 2014). Other studies suggest that late successive bilingualism modulates EFs to a greater extent than balanced bilingualism (Linck et al., 2008; Heidlmayr et al., 2014; Goral et al., 2015). Indeed, L1-dominant bilinguals need to manage asymmetric switch costs because the inhibition of a dominant L1 has been shown to be effortful (Meuter and Allport, 1999; Abutalebi and Green, 2008). This effect should be particularly salient when L1-dominant bilinguals communicate in the second language, which requires the inhibition of the first language. In line with this reasoning, a study by Hernandez and Meschyan (2006) showed that amongst a group of late bilinguals brain areas associated with EFs were activated to a greater extent in a naming task in the second language than in a naming task in the first languages. To investigate the impact of dominance, we assessed the role of individual variability in L1-dominance in this study.

Another important factor related to bilinguals’ dominance pattern is immersion duration. We predicted that our participants will become more balanced and less L1-dominant as a function of increased L2 immersion. Immersion status has been found to modulate not only language abilities, but also the relationship between language and cognitive control processes (Dussias and Sagarra, 2007; Linck et al., 2009; Baus et al., 2013; Beatty-Martínez et al., 2020). Immersion has also been shown to modulate bilinguals’ sociolinguistic habits, with increased L2 immersion favoring greater diversity in bilingual conversation strategies (Beatty-Martínez et al., 2020). Importantly, L2 immersion status may thus be an alternate way through which bilinguals develop high entropy (e.g., Gullifer et al., 2018), favoring a proactive control adjustment to better function in the environment. This issue is also relevant for the monolingual-bilingual comparison as recent research has shown that immersion status may be responsible for differences in cognitive control recruitment strategies (Zirnstein et al., 2018; Navarro-Torres et al., 2019). However, the term “immersion,” i.e., duration of residence in an L2 context, could be argued to be a demographic, rather than a linguistic variable. At the same time, it is of course associated with a range of bilingualism variables, such as shifts in dominance patterns, language usage, etc. To tease apart the relative impact of immersion and its related bilingualism variables on EFs, these factors were entered as separate predictors into the regression model.

### The Present Study

In this study, we investigated the impact of two variables that have been put forward as potential sources of variability in bilinguals’ EF performance: (1) the interactional context in which bilinguals use their languages (monolingual versus bilingual language modes), and (2) bilinguals’ language dominance profiles. To elicit different experimentally-induced language modes, we interspersed a flanker task with either monolingual or bilingual stimuli (whole sentences), adapting an experimental paradigm developed by Wu and Thierry (2013). In the bilingual conditions, we differentiated between different types of intra-sentential code-switching. Five flanker task conditions inducing five language modes were administered: (1) Monolingual (English), (2) Alternational, (3) Insertion of English into German, (4) Insertion of German into English, and (5) Dense code-switching.

All bilingual participants in this study were German–English sequential bilinguals who were immersed in an L2-English context in the United Kingdom. Their L2-immersion had not commenced until after the age of 18. Therefore, they were predicted to be L1-dominant, although they would display different levels of L1-dominance as a function of their duration of immersion. Language switching research suggests that L1-dominant bilinguals experience greater inhibitory cost when suppressing their first language (Meuter and Allport, 1999; Costa and Santesteban, 2004). Hence, for the L1-dominant bilinguals in this study, the inhibitory effort should be greatest in the single-language condition in which the target language is their L2 because of the need to suppress their dominant L1. This was predicted to result in better inhibitory performance in the L2-single-language mode, compared to the bilingual modes. It was also predicted to result in better inhibitory performance when comparing the bilinguals to a monolingual control group who had no second language to suppress.

In the present study, all bilinguals were L1-dominant. The impact of language dominance was investigated by taking a closer look at individual variation within our sample, correlating participants’ degree of L1-dominance with their inhibitory performance in the different interactional contexts. When taking into account individual variation in L1-dominance in our sample, we predicted that the more dominant our bilinguals were in their L1, the better they would perform in the single-language condition. Likewise, the less L1-dominant, and therefore the more balanced, they were, the better they would perform across the bilingual conditions. In addition, the precise nature of control modes triggered by the different bilingual modes was predicted to be modulated by bilinguals’ regular code-switching habits, as assessed in a frequency judgment task. We predicted positive correlations between the frequency with which bilinguals engaged in a given type of code-switching and their performance in the language mode inducing that type of code-switching. Likewise, there should be a negative correlation between the frequency of engaging in a given type of code-switching and performance in language modes inducing non-corresponding control modes, i.e., control modes located at the opposite end of the reactive-proactive spectrum (Figure 1).

An important question in this study was whether engagement in different language modes and code-switching would translate into performance differences between bilinguals and monolinguals. There was no monolingual baseline group in the Wu and Thierry (2013) study, so no conclusions can be drawn on this matter. In the present study, bilinguals’ performance in the single-language mode was compared to that of a monolingual baseline group. It was predicted that the bilinguals would display heightened activation levels of inhibition. The monolinguals, on the other hand, would show no such effect, as they had no need to inhibit a second language. Hence, we predicted that the monolinguals would not display any increased activation of inhibitory control boosting the non-verbal task element. Moreover, the monolinguals do not benefit from any potentially EF-enhancing long-term effects of bilingualism. However, there is a caveat to this prediction. Previous research has shown monolinguals to outperform bilinguals in verbal task conditions (Kharkhurin, 2010). It is therefore possible that monolinguals will show better EF performance in a verbal version of the flanker task. If, on the other hand, bilinguals outperform the monolinguals in a verbal flanker task despite the verbal nature of the task, then this would be a strong indicator for heightened levels of inhibition arising from L1 suppression during the single-language mode.

Previous studies on intra-sentential code-switching and EFs have found positive associations between different code-switching types and performance in associated control modes: alternation has been shown to correlate positively with performance in a flanker task inducing reactive control modes, whilst Dense code-switching correlated positively with inhibitory performance in a flanker task inducing proactive control modes (Hofweber et al., 2016, 2020). However, the observed correlations were based on bilinguals’ regular code-switching practices, as reported in a frequency judgment task. Although frequency judgment tasks are more ecologically valid than questionnaire-based self-reports (Hofweber et al., 2019), they are still mediated by confounds arising from participants’ attitudes to code-switching (Badiola et al., 2018). In this study, we aimed to investigate whether the previously observed interactions between code-switching and EFs can be replicated when eliciting different code-switching modes within the experimental setting itself, and how these effects would correlate with bilinguals’ regular code-switching habits reported in the frequency judgment task.

The phenomenon of fast-modulation of EFs as a result of changes in experimentally-induced language modes raises the question about the extent to which the effects of bilingualism are transient or permanent. On this matter, Wu and Thierry suggest that “bilingual executive control is dependent on fast changing language context rather than long-term language experience” (Wu and Thierry, 2013, p. 13,533). At the same time, Wu and Thierry (2013, p. 13,536) point out that “neuroplastic changes reflect the ‘end product’ of what is usually a long-term experience or training.” If bilinguals are regularly exposed to interactional contexts triggering certain control modes through fast-modulation, then, in the long run, this practice could be hypothesized to lead to more permanently entrenched modulations of the executive system. To date, it remains “unknown whether such advantage is permanent or modulated by the immediate cognitive context” (Wu and Thierry, 2013, p. 13,533). Rather than investigating whether executive control modulations are temporary *or* permanent, posing the research question in an either-or format, this study explored the complex interaction of the effects of habitual and contextual factors on executive control.

To summarize, the design of this study was guided by the following research questions and hypotheses:

RQ 1: To what extent do sequential L1-dominant bilinguals display inhibitory performance differences in the L2-single-language vs. the bilingual conditions of the flanker task?
H 1: Bilinguals are predicted to perform better at inhibition in the L2-single-language than in the bilingual conditions due to heightened levels of inhibition required to suppress the L1.
RQ2: To what extent are there performance differences between monolinguals and bilinguals in the single-language condition of the flanker task?
H2: The L1-dominant bilinguals in this study are predicted to display a boost to inhibitory performance in the L2-single-language condition of the flanker task compared to the monolinguals, as bilinguals will experience heightened levels of inhibitory control due to having to suppress their dominant L1, whilst monolinguals have no need to activate inhibition to suppress another language.
RQ3: To what extent do different bilingual modes (code-switching modes) modulate EF performance in the flanker task?
H3: Code-switching modes involving reactive control (Alternation, Insertion) should lead to better inhibitory performance (measured in the Conflict effect), whilst code-switching modes activating proactive control modes should lead to better monitoring performance (measured in overall RTs).
RQ4: What is the interaction between temporary and permanent effects of bilingualism? How do regular code-switching habits modulate performance in tasks inducing different code-switching modes?
H4: There will be an interaction of fast-modulation effects (temporary transfer effects from language modes) and entrenched bilingualism effects (regular code-switching habits). There will be a positive relationship between the frequency of using a certain type of code-switching and EF performance in the flanker task condition inducing corresponding code-switching control modes. There will be a negative relationship between the frequency of using a certain type of code-switching with EF performance in the flanker task condition inducing non-corresponding code-switching control modes.
RQ5: To what extent does language dominance interact with inhibitory performance in the monolingual and bilingual conditions?
H5: L1-dominance is predicted to correlate positively with inhibitory performance in the L2-single-language mode, and balance (i.e., less L1-dominance) should correlate positively with inhibitory performance in the bilingual modes.

In addition, we predicted the effects of bilingualism on EFs to interact with the effects of participants’ general cognitive abilities (non-verbal IQ, working memory), and with their demographic and linguistic background (age, education, immersion, etc.). These factors will therefore also be explored as potential predictors in the regression analyses, to tease apart the relative effects of bilingualism variables and individuals’ cognitive and socio-economic pre-dispositions.

## Materials and Methods

### Participants

This study included 29 German–English bilinguals and 29 monolinguals. The monolingual group self-reported to be monolingual speakers of English, engaging in no active bilingualism in their everyday lives. Although some participants indicated having taken foreign language classes in French, German, or Spanish in secondary school, they had never used these languages in daily life, and stopped learning after graduation. Hence, they were considered to be functionally monolinguals (Anderson et al., 2018). The bilinguals and monolinguals were carefully matched on a range of variables that impact EF performance, i.e., Age, Education, non-verbal IQ, Working-, and Short-term memory (Table 1). EFs have been proven to be particularly prone to Age effects, due to the effects of cognitive maturation and subsequent age-related decline (Dempster, 1992). It is widely reported that older adults experience a decline of EF abilities during both linguistic and non-linguistic processing, including reduced information processing speed and reduced inhibitory capacity (Salthouse and Meinz, 1995). In this study, the age range of participants was not restricted. A level of variability within each group was in fact intended because we operationalized participants’ demographic background variables as continuous variables, to be able to observe the effects of individual differences in linear models. For the purpose of the group comparison of bilinguals and monolinguals, the ages of participants in the two groups were matched in terms of both central tendencies and range.

**TABLE 1:** Non-linguistic background variables.

		Monolinguals	Bilinguals	*F*-value	df	*p*-value
**Age**	Mean	31.25	34.21	0.88	1, 55	0.35
	*SD*	13.30	10.44			
	Range	17.00–69.00	22.00–71.00			
**Education**	Mean	4.18	4.31	0.32	1, 55	0.57
	*SD*	0.48	1.14			
	Range	4.00–6.00	1.00–6.00			
**Non-verbal IQ**	Mean	112.04	113.28	0.12	1, 55	0.74
	*SD*	10.92	16.05			
	Range	95.00–145.00	75.00–145.00			
**Short term memory**	Mean	6.61	6.48	0.23	1, 55	0.64
	*SD*	1.13	0.88			
	Range	5.00–9.00	5.50–9.00			
**Working memory**	Mean	4.61	4.59	0.00	1, 55	0.95
	*SD*	1.31	0.85			
	Range	3.00–9.00	3.00–7.00			

Whilst in a previous study we had investigated the interaction between code-switching and EFs by comparing speakers with the same language combination in different sociolinguistic contexts (Hofweber et al., 2016), this study focused on assessing individual differences as predictors of EFs, whilst keeping the sociolinguistic background of participants “constant.” All participants were German–English bilinguals who were first generation immigrants to the United Kingdom. To assess individual differences in bilinguals’ language profiles, an online Language history questionnaire (Li et al., 2014) was used to collect general demographic and linguistic background information, such as Age of Onset of the L2, Proficiency, and Immersion (duration of stay in the L2 environment). We focused on assessing bilinguals’ language dominance patterns, which may modulate the effect of bilingualism on EFs (Treffers-Daller, 2016).

The term language dominance is frequently used in bilingualism studies, but is not always well-defined and measured based on a clear rationale (Silva-Corvalan and Treffers-Daller, 2016). In this study, dominance was operationalized using two strategies. Firstly, it was computed as the relative difference in proficiencies between the two languages (Kupisch and Van de Weijer, 2016). Secondly, we administered Dunn and Fox Tree’s (2009) Bilingual dominance scale, which conceptualizes dominance as a multi-component construct. To account for the complexity of the language dominance variable, we therefore also assessed language balance as measured in the Bilingual dominance scale, which generates dominance scores for each language on an interval scale. The dominance scale questionnaire is based on 12 questions asking participants about issues associated with the notion of dominance. Participants are asked to indicate their age of onset, their language usage preference (at home, when doing mental maths), their accent, their schooling and their fluency in each respective language. A scoring manual allows for the computation of an overall dominance score for each language.

We predicted that bilinguals in this study would be dominant in the L1 German because they started learning the L2 English with a late Onset Age (*M* = 9.83, *SD* = 4.26). Moreover, participants’ L2-immersion only began in adulthood, after the age of 18. Indeed, a repeated-measures ANOVA with the within-subject variable Dominance (German, English) revealed that bilinguals’ Dominance score for German (*M* = 20.25, *SD* = 3.68) was significantly higher [*F*(1,27) = 39.99, *MSE* = 24.45, *p* < 0.00001, η^2^ = 0.60] than their Dominance score for English (*M* = 11.89, *SD* = 4.64). Bilinguals’ L1-dominance thus persisted despite their L2-immersion. Nevertheless, there was individual variability in the data set: bilinguals’ dominance pattern shifted as a function of immersion, with longer immersed bilinguals becoming more balanced and less L1-dominant [*R*(1,27) = −0.33, *p* = 0.02]. Table 2 presents an overview of the linguistic background variables.

**TABLE 2:** Linguistic background variables.

		Bilinguals
**Proficiency German**	Mean	6.92
	*SD*	0.33
	Range	5.25–7.00
**Proficiency English**	Mean	6.56
	*SD*	0.48
	Range	5.50–7.00
**Balance (German–English proficiency)**	Mean	0.51
	*SD*	0.75
	Range	0.00–3.06
**Age of onset English**	Mean	9.83
	*SD*	4.26
	Range	0.00–27.00
**Immersion in years**	Mean	11.83
	*SD*	10.91
	Range	1.00–48.00
**Dominance German (Dunn and Fox Tree, 2009)**	Mean	20.25
	*SD*	3.68
	Range	14.00–27.00
**Dominance English (Dunn and Fox Tree, 2009)**	Mean	11.89
	SD	4.64
	Range	5.0–24.00

### Assessing the Independent Variable Code-Switching Habits

To measure the bilinguals’ code-switching habits, they were presented with 14 code-switches of each type (Alternation, Insertion English into German, Insertion German into English, Dense code-switching) and were asked to provide frequency judgments of their usage of the different code-switching types on a 7-point Likert scale (rating scale: 1 = “never use” to 7 = “use all the time”). The code-switching stimuli were authentic utterances sourced from existing corpora of code-switching in this language pair, i.e., a corpus of German L1 speakers who emigrated to the United Kingdom in the 1930s (Eppler, 2005, 2010), and a group of German L1 heritage speakers residing in Australia (Clyne, 2003). Moreover, our source materials comprised a set of bilingual emails collected for a previous study with German–English bilinguals in the United Kingdom and German heritage speakers in South Africa (Hofweber et al., 2016, 2019). The code-switching utterances were presented in both the written and the auditory format. The stimulus presentation and response time was limited to 30 s, after which the next trial would appear. A more detailed description of the frequency judgment task design, and a discussion of the validity of frequency judgment tasks in assessing bilinguals’ code-switching habits can be found in Hofweber et al. (2019).

### Assessing the Dependent Variable Executive Control

To measure inhibitory control, we administered a flanker task. In each trial, participants were presented with a horizontal row of five arrows and were instructed to indicate the direction of the central arrow by a key press (left arrows key for left-facing keys, right arrows key for right-facing arrows). In each condition, there were 48 “congruent” trials, in which all arrows faced in the same direction. These were contrasted with 48 “incongruent” trials, in which the distractor arrows faced in the opposite direction, compared to the target arrow. To give the correct response in incongruent trials, participants needed to recruit inhibitory control to suppress the directionality of the distractors. The performance difference (RTs and accuracy) between congruent and incongruent trials thus measures inhibitory load. It is labeled as the “Conflict effect.” The split between congruent and incongruent trials was 50:50. This means that participants continuously needed to switch between congruent and incongruent trials, creating a high-monitoring context (Costa et al., 2009; Hofweber et al., 2016). This is the version of the flanker task in which previous research has identified effects of bilingualism (Costa et al., 2009). As we were interested in investigating what underlies these previously observed effects, we chose to administer this version of the flanker task.

In this study, we were interested in observing inhibitory performance in different language modes. To induce these language modes, we adopted an experimental paradigm developed by Wu and Thierry (2013), in which flanker trials were interspersed with either monolingual or bilingual stimuli. The aim of the verbal manipulation of the flanker task was to activate control modes associated with different language modes. We administered five conditions of the flanker task, inducing the following language modes: single-language (English), Alternation, Insertion (English into German), Insertion (German into English). Each condition included 96 trials (48 congruent, 48 incongruent). To avoid unintended order effects, the task blocks were presented in a partially counterbalanced order. The monolingual group only took the single language verbal version of the flanker task. The monolinguals were administered the flanker task as part of a slightly different experimental battery, which generated data for monolingual baseline comparisons in both this study and the Hofweber et al. (2020) study. This meant that they first completed a set of three non-verbal flanker task conditions (96 trials in each condition), before moving on to the verbal flanker task interspersed by sentences, so they consistently took the verbal flanker task as the fourth task block. If anything, they should therefore have an advantage over the bilinguals as they will have experienced a greater practice effect on average. Nevertheless, this is a limitation for a direct group comparison.

In the code-switching conditions, the verbal stimuli contained the relevant type of code-switching. The code-switching utterances were sourced from existing corpora of German–English bilingual speech (Clyne, 2003; Eppler, 2005) and classified using a detailed catalog of criteria devised by Deuchar et al. (2008). All verbal stimuli were presented in the written format. Code-switches were marked in bold letters, marking the switch points. This was intended to be analogous to transitions in phonology, which mark code-switching in spoken language. Participants did not have to react to the verbal stimuli, but they were told to read the utterances thoroughly as there would be questions about them at the end. Unbeknown to the participants, there were no questions at the end. The instruction was only given to make sure participants actually read, and therefore processed the presented stimuli. This study, thus, differs from Wu and Thierry’s (2013) study, in which participants were not explicitly instructed to read the word stimuli.

**FIGURE 2:**
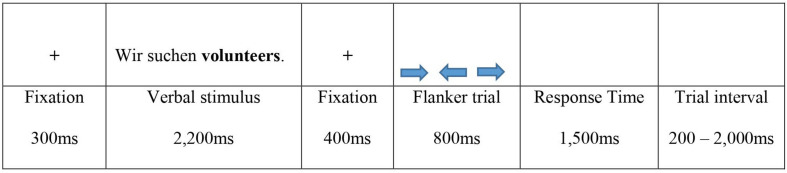
Individual trial verbal flanker task.

The order and duration of the presentation of stimuli followed Wu and Thierry’s approach, with the exception of some minor adjustments (Figure 2). Wu and Thierry’s (2013) individual word stimuli were replaced with stimuli containing full sentences. To allow participants to process this more complex information, the duration of presentation of the verbal stimuli was increased from 1,500 to 2,200 ms. The verbal stimuli were preceded by a 300 ms fixation cross and the flankers by a fixation cross of 400 ms. Each flanker stimulus was shown for 800 ms, and was then followed by a blank screen, allowing an additional maximal response time of 1,500 ms. Trial intervals were jittered from 200 to 2,000 ms.

## Results

### Code-Switching Patterns Revealed by the Frequency Judgment Task

**FIGURE 3:**
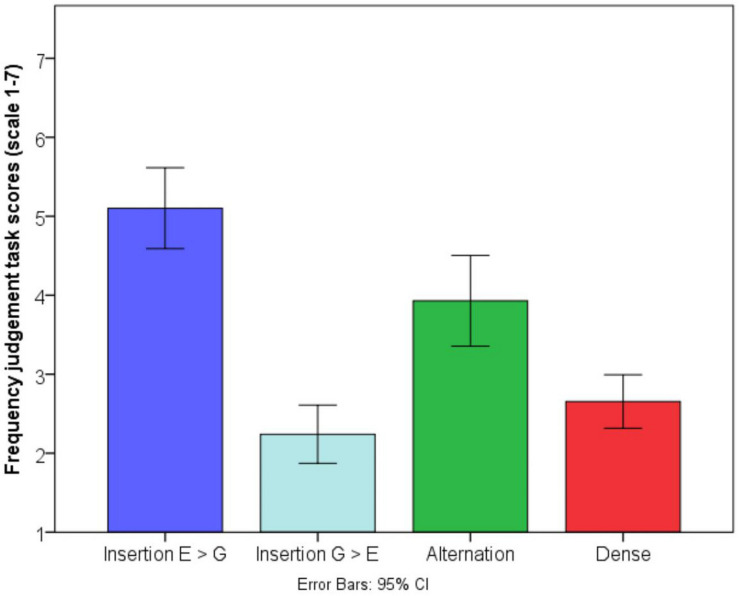
Frequency judgment task scores.

The German–English bilinguals in this study engaged in all types of code-switching to some extent (Table 3 and Figure 3). To assess differences between their frequency of use of the four code-switching types, we conducted a within-subjects ANOVA with Code-switching type (Insertion English into German, Insertion German into English, Alternation, Dense code-switching) as the within-subjects variable and frequency scores from the judgment task (1–7) as the dependent variable. There was a significant effect of code-switching type, i.e., the frequency scores across the four code-switching types differed [*F*(3,84) = 82.66, *MSE* = 0.59, *p* < 0.001, η^2^ = 0.75)]. The most frequently practiced code-switching type was Insertion English into German (*M* = 5.10, *SD* = 1.35), followed by Alternation (*M* = 2.24, *SD* = 0.97). The least frequently practiced code-switching type was Dense code-switching (*M* = 2.65, *SD* = 0.89) and Insertion German into English (*M* = 2.24, *SD* = 0.97). Frequency of Insertion English into German was significantly greater than all other code-switching types at the *p* < 0.001 level. Alternational code-switching frequency was also greater than frequency of Insertion German into English (*p* < 0.001) and frequency of Dense code-switching (*p* < 0.001). However, Insertion German into English and Dense code-switching were given equally low frequency scores (*p* = 0.17). Dense code-switching occurred only infrequently amongst our participants because they were first-generation immigrants, and Dense code-switching occurs predominantly in closely-knit multilingual communities with long-standing bilingual traditions (Muysken, 2000; Hofweber et al., 2016). Insertion of German into English is uncommon because German tends to be the matrix language when German-dominant bilinguals converse with each other. This distribution is also consistent with previous findings by Hofweber et al. (2016, 2019, 2020) for German–English bilinguals with a similar sociolinguistic profile, i.e., 1st generation immigrants to the United Kingdom who are loosely connected through communities of practice, rather than closely knit speech communities. Bilinguals’ overall frequency of code-switching (average of frequency reported for all types of code-switching) correlated positively with each separate type of code-switching [Insertion G > E: *R*(1,29) = 0.75, *p* < 0.01; Insertion E > G: *R*(1,29) = 0.86, *p* < 0.01; Alternation: *R*(1,29) = 0.94, *p* < 0.01; *R*(1,29) = 0.82, *p* < 0.01], suggesting that those who code-switched frequently did so across all types of code-switching.

**TABLE 3:** Frequency judgment task scores.

		Bilinguals
**Insertion E > G**	Mean	5.10
	*SD*	1.35
	Range	1.90–6.86
**Insertion G > E**	Mean	2.24
	*SD*	0.97
	Range	1.00–4.64
**Alternation**	Mean	3.93
	*SD*	1.51
	Range	1.14–6.50
**Dense code-switching**	Mean	2.65
	SD	0.89
	Range	1.14–4.50

### Flanker Task Performance

For the RT analyses, we included values within three SDs of the mean. Participants’ average RTs were distributed normally (*K*–*S* test: *p* > 0.05), so parametric tests could be used. Across all analyses, Mauchly’s test of sphericity was significant (*p* < 0.05); therefore the numbers presented here are based on Greenhouse–Geisser corrections. Participants performed close to ceiling on Accuracy. As a result, the Accuracy rate distribution was strongly skewed, resulting in a non-normal distribution (*K*–*S* test: *p* < 0.0001). Therefore, non-parametric tests were used to assess Accuracy. Previous studies have reported a low internal validity for Flanker tasks (Von Bastian et al., 2016), so we assessed our task’s split-half reliability. In this study, the reliability was fairly high for RTs (congruent: *Spearman’s rho* = 0.89, *p* < 0.01; incongruent: *Spearman’s rho* = 0.86, *p* < 0.01), although Accuracy converged only on congruent trials (congruent: *Spearman’s rho* = 0.39, *p* < 0.05; incongruent: *Spearman’s rho* = 0.08, *p* > 0.05).

#### Comparison of Bilinguals’ Executive Performance in the Single-Language Mode and in the Bilingual Modes

We predicted that the L1-dominant sequential bilinguals in this study would perform better in the single-language mode than in the bilingual modes due to heightened levels of inhibition. To allow for a direct comparison of executive performance in single-language versus bilingual modes, we collapsed the average performance across all bilingual conditions, thus generating overall “bilingual mode” performance scores for congruent and incongruent trials. Then, we conducted a repeated-measures ANOVA with Language mode (single-language, bilingual) and Congruency (congruent, incongruent) as the within-subjects variables and RTs as the dependent variable. The effect of Congruency was significant [*F*(1,28) = 337.32, *MSE* = 294.39, *p* < 0.0001, η^2^ = 0.92], generating the expected Conflict effect typical of the flanker task paradigm: RTs in congruent trials (*M* = 496.87 ms) were significantly (*p* < 0.0001) shorter than in incongruent trials (*M* = 555.38 ms). Importantly, the analysis revealed a main effect of Language mode [*F*(1,28) = 9.27, *MSE* = 740.19, *p* < 0.01, η^2^ = 0.25]. Bilinguals had shorter RTs in the single-language mode (*M* = 518.43 ms) than in the bilingual modes (*M* = 533.82 ms), the difference being strongly significant (*p* < 0.01). The interaction between Congruency and Language mode was not significant [*F*(1,28) = 0.05, *MSE* = 121.71, *p* = 0.83, η^2^ = 0.002].

Friedman tests were conducted to compare Accuracy performance at congruent and incongruent trials in the monolingual and bilingual modes. In congruent trials there was no difference (Chi-square = 0.33, *p* = 0.56) in Accuracy between the single-language mode (*M* = 99.57%, *SD* = 1.02%) and the bilingual modes (*M* = 99.72%, *SD* = 0.43%). In incongruent trials, bilinguals performed significantly better (Chi-square = 10.67, *p* < 0.001) in the monolingual (*M* = 99.50%, *SD* = 1.20%), compared to the bilingual modes (*M* = 99.10%, *SD* = 0.87%). This means that the single-language mode appeared to enhance Accuracy performance in trials requiring inhibitory control. Bilinguals thus displayed a significantly greater (Chi-square = 7.35, *p* < 0.01) Conflict effect in the bilingual modes (*M* = 0.62%, *SD* = 1.56%), compared to the single-language mode (*M* = 0.07%, *SD* = 1.18%). This means that the monolingual block did not only yield reduced RTs, but also generated better inhibitory performance.

#### Comparison of Monolinguals’ and Bilinguals’ Executive Performance

The second research question concerned differences between the monolinguals and bilinguals in the single-language condition of the flanker task. Table 4 shows the bilinguals’ and monolinguals’ RTs in this condition. To compare the performance of bilinguals and monolinguals in the single-language condition, we conducted a mixed-design ANOVA with Congruency (congruent, incongruent) as the within-subjects variable and Group (monolingual, bilingual) as the between-subjects variable. This showed a significant effect of Congruency [*F*(1,56) = 386.45, *MSE* = 233.98, *p* < 0.001, η^2^ = 0.87] with congruent trials yielding shorter RTs than incongruent trials. The between-subjects comparison showed no reliable differences [*F*(1,56) = 1.92, η^2^ = 0.03, *p* = 0.17] and there was no significant interaction between Group and Congruency either [*F*(1,56) = 0.62, *p* = 0.43, η^2^ = 0.01]. Therefore, monolinguals and bilinguals did not perform differently at RTs overall or at congruent and incongruent trials specifically. Table 5 shows the bilinguals’ and monolinguals’ Accuracy in the monolingual block. Friedman tests were conducted to explore the effect of Congruency in each group separately, and Mann–Whitney *U*-tests were used to explore between-subjects differences. The within-subjects comparison of congruent and incongruent trials revealed a Congruency effect in the monolingual group [Chi-square (1,29) = 6.23, *p* < 0.01], but not in the bilingual group [Chi-square (1,29) = 0.00, *p* = 1.00]. As can be seen from Table 5, the between-subjects comparison showed that bilinguals and monolinguals performed equally well at congruent trials (Mann–Whitney *U* = 418.50, *p* = 0.96), but that there was a trend for bilinguals to perform more accurately than monolinguals on incongruent trials (Mann–Whitney *U* = 328.00, *p* = 0.07). Specifically, bilinguals outperformed monolinguals on the measure of inhibition, the Conflict effect (Mann–Whitney *U* = 302.50, *p* = 0.028). In fact, bilinguals experienced hardly any Conflict effect at all, whilst monolinguals experienced a classic conflict effect. This means that whilst the monolinguals made significantly more errors in the trials requiring inhibitory effort, such increased inhibitory effort did not lead to an increase in errors in the bilingual group.

**TABLE 4:** RTs in the single-language condition.

RTs in ms		Monolinguals	Bilinguals	*F*-value	df	*p*-value
**Single-language congruent**	Mean	475.02	489.39	0.14	1, 56	0.71
	*SD*	44.50	43.82			
	Range	375.45–561.82	423.83–557.65			
**Single-language incongruent**	Mean	528.61	547.48	0.37	1, 56	0.55
	*SD*	48.04	50.99			
	Range	406.16–628.82	480.84–681.39			
**Single-language Conflict effect**	Mean	52.19	56.74	0.56	1, 56	0.46
	*SD*	21.46	24.80			
	Range	12.88–98.28	19.09–134.19			

**TABLE 5:** Accuracy rates in the single-language condition.

Accuracy in %		Monolinguals	Bilinguals	Mann–Whitney *U*	*p*-value
** Congruent**	Mean	99.81	99.78	418.50	0.96
	*SD*	0.49	0.51		
	Range	97.92–100.00	97.92–100.00		
**Incongruent**	Mean	99.12	99.75	328.00	0.07
	*SD*	1.64	0.60		
	Range	92.71–100.00	97.92–100.00		
**Conflict effect**	Mean	0.69	0.04	302.50	*0.03
	*SD*	1.60	0.59		
	Range	−2.08 – 6.25	−1.04 – 2.08		

*p < 0.05.

#### Bilinguals’ Executive Performance in the Different Bilingual Code-Switching Conditions

Table 6 shows the bilingual participants’ RTs in the five language mode conditions. To address the third research question, i.e., whether bilinguals displayed differences in EF performance across the different code-switching modes we conducted a repeated-measures ANOVA with Condition (Monolingual, Alternation, Insertion English into German, Insertion German into English, Dense code-switching) and Congruency (congruent, incongruent) as the within-subjects variables, and RTs as the dependent variable. There was a strongly significant effect of Congruency [*F*(1,28) = 400.49, *MSE* = 625.46, *p* < 0.0001, η^2^ = 0.94]. Incongruent trials yielded greater RTs (*M* = 560.13) than congruent trials (*M* = 501.35), so the experimental manipulation generated the intended Conflict effect. When assessing the impact of the language mode condition on RTs, the analysis revealed that the effect of Condition [*F*(1,87,52.39) = 3.12, *MSE* = 5559.88, *p* = 0.056, η^2^ = 0.10] was marginally significant, i.e., there was a trend for RTs to differ across the five task conditions.

**TABLE 6:** RTs in the different conditions.

RTs in ms		Congruent trials	Incongruent trials	Conflict effect
**Single-language**	Mean	489.39	547.48	58.08
	*SD*	43.82	50.99	23.72
	Range	423.83–557.65	480.84–681.39	22.10–134.19
**Alternation**	Mean	502.59	563.91	61.30
	*SD*	50.90	55.45	17.95
	Range	432.65–599.06	488.18–665.29	31.56–101.15
**Insertion E > G**	Mean	502.36	569.36	67.00
	*SD*	52.56	69.62	31.17
	Range	429.95–615.59	477.92–754.25	33.17–186.86
**Insertion G > E**	Mean	492.19	546.98	54.80
	*SD*	48.55	52.81	16.99
	Range	423.22–584.24	475.55–646.11	32.72–91.77
**Dense**	Mean	520.21	572.92	52.70
	*SD*	82.72	82.25	26.36
	Range	430.69–843.66	488.62–850.67	0.00–133.18

Importantly, there was a marginally significant interaction between Condition and Congruency [*F*(2.89,80.85) = 2.33, *MSE* = 639.84, *p* = 0.08, η^2^ = 0.08], suggesting that there was a trend for the Congruency pattern to differ across the three conditions, or for the Condition effect to differ in congruent compared to incongruent trials. Paired comparisons using Bonferroni adjustments for multiple comparisons were conducted to investigate this interaction further. This analysis showed that incongruent trials reliably yielded greater RTs than congruent trials (*p* < 0.0001) across all five blocks. However, there were no significant differences for RTs in congruent trials across the five blocks. Incongruent trials displayed the highest RTs in the Dense block (*M* = 572.92 ms, *SD* = 82.25 ms), followed by Insertion of English into German (*M* = 569.36 ms, *SD* = 69.62 ms) and Alternation (*M* = 563.91 ms, *SD* = 55.45 ms). In line with results from congruent trials, the monolingual English mode (*M* = 547.48 ms, *SD* = 50.99 ms) and Insertion of German into English (*M* = 546.98 ms, *SD* = 52.81 ms) yielded the lowest incongruent RTs. In the case of incongruent trials, there was a significant difference between Alternation and Insertion of German into English (*p* = 0.047), as well as a marginally significant difference between the two types of Insertion (*p* = 0.084). None of the remaining differences between language modes were significant.

To summarize, there was a trend for RTs to be greatest in the Dense code-switching condition. Those conditions using the L2 English as the only language (Monolingual) or as the main matrix language (Insertion German into English) yielded the lowest RTs. In congruent trials, differences between conditions were not significant, but in incongruent trials, there was a trend for the differences between conditions to reach significance.

Non-parametric Friedman tests were used to assess within-subjects variation for Accuracy (Table 7). In congruent trials, there was no difference between the five blocks [Chi-square (4) = 1.34, *p* = 0.86]. In the incongruent trials, there was a trend for Accuracy to differ across the language blocks (Chi-square = 8.27, *p* = 0.08). Accuracy was highest in the Monolingual block (*M* = 99.50%, *SD* = 1.20%), followed by the Alternational (*M* = 99.43%, *SD* = 1.23%) and Dense code-switching blocks (*M* = 99.43%, *SD* = 1.10%). The Insertional blocks yielded slightly lower Accuracy rates (Insertion English into German: *M* = 98.71%, *SD* = 1.52%, Insertion German into English: *M* = 98.71%, *SD* = 2.19%). When conducting Friedman comparisons for each condition pairwise, the only significant difference occurred between Accuracy in the Monolingual block and Accuracy in the Insertional (English into German) block (Chi-square = 5.40, *p* = 0.02).

**TABLE 7:** Accuracy rates in the different conditions.

Accuracy in %		Congruent trials	Incongruent trials	Conflict effect
**Single-language**	Mean	99.57	99.50	0.07
	*SD*	1.02	1.20	1.18
	Range	96.00–100.00	96.00–100.00	−2.00–4.00
**Alternation**	Mean	99.71	99.43	0.28
	*SD*	0.73	1.23	1.16
	Range	98.00–100.00	96.00–100.00	−2.00–4.00
**Insertion E > G**	Mean	99.64	98.71	0.90
	*SD*	0.80	1.52	1.74
	Range	98.00–100.00	96.00–100.00	−2.00–4.00
**Insertion G > E**	Mean	99.78	98.71	1.03
	*SD*	0.65	2.19	2.31
	Range	98.00–100.00	94.00–100.00	−2.00–6.00
**Dense**	Mean	99.71	99.55	0.28
	*SD*	0.92	0.85	1.03
	Range	96.00–100.00	98.00–100.00	−2.00–2.00

#### Predictors of Inhibitory Performance in the Single-language Flanker Task Condition

Research questions 4 and 5 were concerned with the predictors of inhibitory performance in the different language modes. We first investigated the predictors of inhibitory performance in the single-language mode, in which performance differences between monolinguals and bilinguals had occurred. A stepwise multiple regression was conducted for monolinguals and bilinguals separately. The following non-linguistic predictor variables were used: Age, non-verbal IQ, Education, Short-term memory, Working memory. As outcome variables, we focused on the measures of inhibitory performance, i.e., RTs and Accuracy in incongruent trials and the Conflict effect (cf. Table 8 for a summary of significant predictors).

**TABLE 8:** Summary of predictors in the regression single-language condition.

Predictors regression	Monolinguals	Bilinguals
**Conflict effect (accuracy)**	WM	
**Conflict effect (RTs)**		Dense CS
**Incongruent trials (accuracy)**	WM	Dense CS
**Incongruent trials (RTs)**	Age	STM, IQ

##### Reaction times

In incongruent trials, Age explained 26.6% of RT performance variance in the monolingual group [*R*(1,26) = 0.52, *R* square = 0.27, adj. *R* square = 0.24, *B* = 1.84, β = 0.52, *Constant* = 473.56, *F*-change = 9.44, *p* < 0.01], and Short-term memory and IQ explained 45.7% of RT variance in the bilingual group [*R*(1,26) = 0.68, *R square* = 0.46, adj. *R* square = 0.42, Short-term memory: *B* = −29.89, β = −0.52, IQ: *B* = −1.27, β = −0.40, *Constant* = 473.56, *F*-change = 7.54, *p* < 0.01]. The regression with the outcome variable Conflict effect measured in RTs revealed no significant predictor variables. Inhibitory control performance thus remained unexplained by non-linguistic predictors, which called for further analyses using linguistic predictors in the bilingual group.

##### Accuracy

In monolinguals, Working memory explained the Conflict-effect measured in Accuracy [*R*(1,26) = 0.46, *R square* = 0.21, adj. *R* square = 0.18, *B* = 0.01, β = 0.46, *Constant* = −0.04, *F*-change = 6.83, *p* = 0.02] as well as Accuracy in incongruent trials [*R*(1,26) = 0.46, *R square* = 0.21, adj. *R* square = 0.18, *B* = −0.01, β = −0.46, *Constant* = 1.04, *F*-change = 7.02, *p* < 0.01]. In the bilingual group, none of the non-linguistic predictors explained performance variance at Accuracy. However, it was in Accuracy measures of inhibitory control that bilinguals outperformed monolinguals. It is therefore of particular interest to better understand predictors of bilinguals’ inhibitory performance in this condition. This prompted further analyses using linguistic predictor variables.

To investigate whether linguistic predictors could explain bilinguals’ performance in the single-language condition, the following variables were entered into a stepwise regression: Proficiency, English Age of Onset, Balance, Immersion (duration of residence in the L2 context), Code-switching frequency scores (Insertion English into German, Insertion German into English, Alternation, Dense code-switching).

##### Reaction times

**FIGURE 4:**
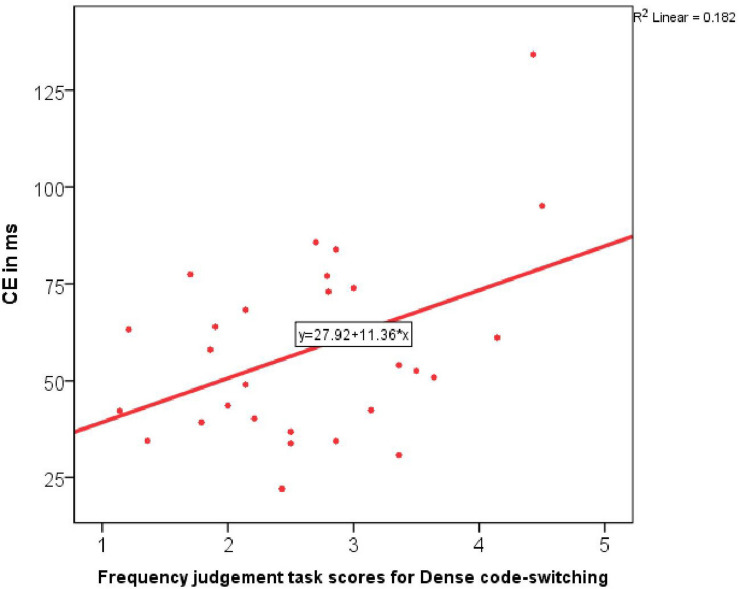
Correlation between the Conflict effect in the monolingual task block and Dense code-switching frequency.

None of these variables predicted bilingual RTs in incongruent trials. However, when it came to predicting the actual measure of inhibition, the Conflict effect, the stepwise regression identified one significant predictor and that was Dense code-switching frequency [*R*(1,27) = 0.43, *R square* = 0.182, adj. *R square* = 0.15, *B* = 11.36, β = 0.43, *Constant* = 27.92, *F*-change = 6.01, *p* = 0.02]. The more frequently participants engaged in Dense code-switching, the greater was their Conflict effect, i.e., the less well they performed at inhibition in the single-language condition (Figure 4).

##### Accuracy

**FIGURE 5:**
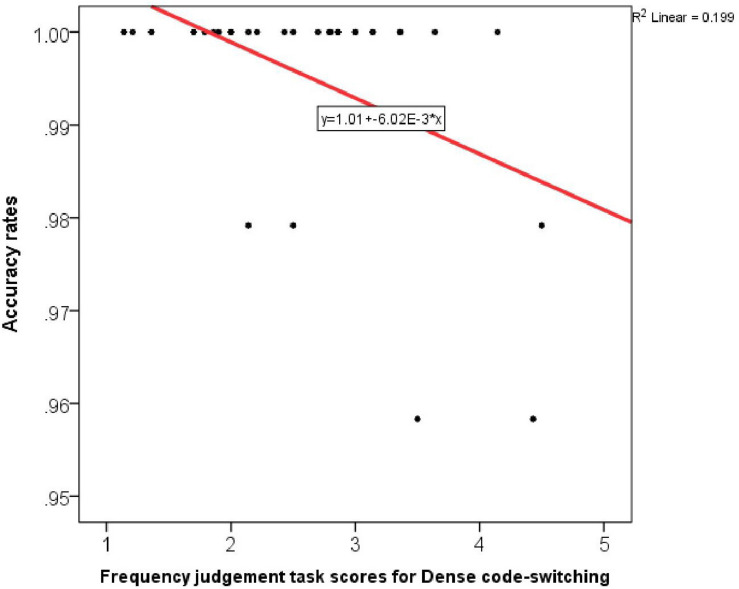
Correlation between Accuracy rates in incongruent trials in the single-language condition and Dense code-switching frequency.

The conflict effect for Accuracy was not predicted by any of the linguistic variables. However, Accuracy performance in incongruent trials was also predicted negatively by Dense code-switching, which explained 19.80% of the variance in Accuracy [*R*(1,26) = 0.45, *R square* = 0.34, adj. *R square* = 0.20, *B* = −0.01, β = −0.45, *Constant* = 1.01, *F*-change = 6.44, *p* = 0.02]. The more frequently bilinguals reported to Densely code-switch, the more errors they made in incongruent trials (Figure 5). When predicting inhibitory performance, Dense code-switching therefore was a negative predictor of bilingual inhibitory performance in the single-language condition. This would suggest that the control modes trained by Dense code-switching (proactive control modes) do not correspond with the control modes activated by the single-language mode (reactive control modes).

However, this observation needs to be treated with caution. As can be seen from Figure 5, most participants performed at ceiling, so this correlation is mainly driven by the five cases in which Accuracy was slightly below 100%. Although these five items were not identified as outliers in the case-wise diagnostics and all of them coincided with high Dense code-switching scores, we cannot really draw a reliable conclusion from these results.

#### Predictors of Inhibitory Performance in the Bilingual Flanker Task Conditions

In research questions 4 and 5, we predicted the following factors to influence performance in the different language modes: bilinguals’ regular code-switching habits, bilinguals’ language dominance profiles, bilinguals’ general cognitive abilities. To identify predictor variables of performance in the different language mode conditions in the flanker task amongst the bilingual group, and to tease apart the relative impact of linguistic and non-linguistic factors on executive performance, a combination of stepwise and hierarchical regressions was conducted. Initially, exploratory stepwise regressions were conducted to isolate variables that are candidates for being significant predictors. The first stepwise regression was conducted with seven non-linguistic predictor variables: IQ, Age, Education, Short term memory English, Short term memory German, Working memory English, Working memory German. The second stepwise regression was conducted with the following linguistic predictor variables: Code-switching frequency scores from the judgment task for Insertion German into English, Insertion English into German, Dense code-switching, as well as English Age of Onset, English language proficiency, Balance, Immersion. The linguistic and non-linguistic variables identified as significant predictors in the two initial stepwise regressions were subsequently entered into two types of hierarchical regression models, one entering the non-linguistic variables as control variables and the linguistic ones as predictor variables, and another one entering the linguistic variables as control variables and the non-linguistic variables as predictor variables. The following sections present the results obtained from this procedure. Due to the complexity of the procedure, we are only presenting the models created for the dependent variable Conflict effect measuring inhibitory control, expressed in both RTs and Accuracy.

##### Predictors of performance in the alternational code-switching mode

**FIGURE 6:**
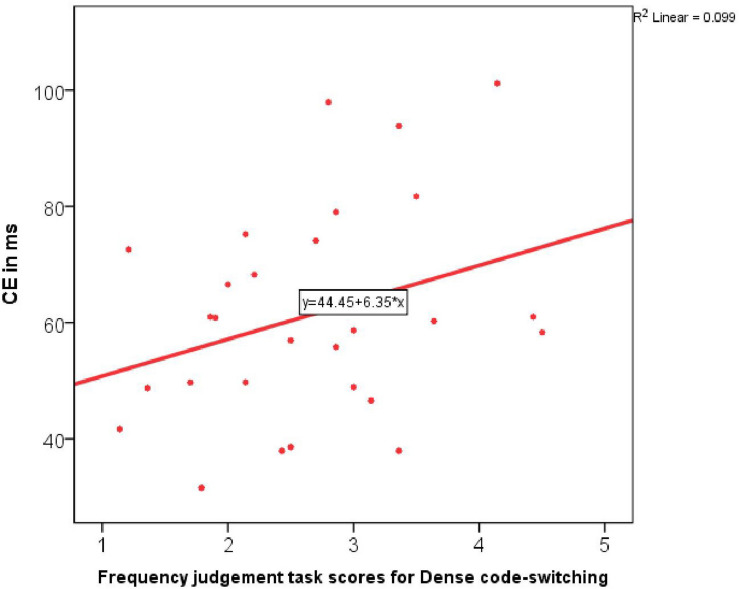
Correlation between the Conflict effect in the Alternational task block and Dense code-switching frequency.

In the condition inducing an Alternational code-switching mode, the Conflict effect measured in RTs was best explained by a model based on Insertion of German into English as the primary variable and Working memory as the control variable [*R*(1,26) = 0.55, *R square* = 0.30, adj. *R square* = 0.24, Insertion G > E: *B* = 5.37, β = 0.29, WME: *B* = −6.35, β = −0.38, *Constant* = 79.20, *F*-change = 4.67, *p* = 0.04]. Bilinguals who engaged more frequently in Insertion of German into English performed less well at the type of inhibitory control associated with the Alternational code-switching block. This performance was modulated by Working memory abilities, which enhanced inhibitory performance. Interestingly, although Dense code-switching was not singled out as a significant predictor in the regression analysis, the correlation matrix flagged a significant positive correlation between Dense code-switching and the Conflict effect (*R* = 0.32, *p* = 0.048). This means that the more frequently bilinguals engaged in the proactive control modes associated with Dense code-switching, the less well they performed in the Alternational condition requiring the activation of reactive control modes (Figure 6). To summarize, inhibitory performance was predicted negatively by Insertion German into English, suggesting that this type of Insertion engages inhibitory mechanisms different from those activated in the Alternational code-switching mode. There was also a negative correlation between Dense code-switching frequency and inhibitory performance in the Alternational block. None of the non-linguistic variables predicted Accuracy rates in either the congruent or incongruent trials or the Conflict effect measured in Accuracy.

##### Predictors of performance in the insertion E > G mode

In the analyses of inhibitory performance measured in the Conflict effect (RTs), the best-fitting model turned out to be the one based on Working memory as the primary predictor and Balance and Proficiency as control variables [*R*(1,25) = 0.81, *R square* = 0.66, adj. *R square* = 0.62, WME: *B* = −6.17, β = −0.21, Balance: *B* = 37.34, β = 0.52, Proficiency: *B* = 33.76, β = 0.52,*Constant* = −144.19, *F*-change = 18.82, *p* < 0.0001]. In this model Working memory explained 14.2% of inhibitory performance variance and the linguistic variables Balance and Proficiency another 51.6%. When assessing the Conflict effect measured in Accuracy rates, Alternation was a positive predictor of the size of the Conflict effect [*R*(1,26) = 0.49, *R square* = 0.24, adj. *R square* = 0.21, *B* = 0.006, β = 0.49, *Constant* = −1.30, *F*-change = 8.24, *p* < 0.01]. This suggests that the more frequently bilinguals engaged in Alternational code-switching, the less well they performed at inhibition in the flanker task block assumed to induce an Insertional code-switching mode.

##### Predictors of performance in the insertion G > E mode

The hierarchical regressions taking into account both linguistic and non-linguistic variables show that the best explanatory model of the Conflict effect (RTs) comprised Balance as the primary predictor and Working memory as the control variable [*R*(1,25) = 0.69, *R square* = 0.48, adj. *R square* = 0.42, Balance: *B* = 6.42, β = 0.28, WMG: *B* = 8.05, β = 0.41, WME: *B* = −11.16, β = −0.70, *Constant* = 7171.22, *F*-change = 7.92, *p* < 0.01]. In this model Balance accounted for 15% of inhibitory performance variance and Working memory for 33%. More balanced bilinguals performed better at inhibition in this condition. The Conflict effect measured in accuracy rates was explained by Alternational code-switching frequency [*R*(1,26) = 0.45, *R square* = 0.21, adj. *R square* = 0.18, *B* = 0.007, β = 0.45, *Constant* = −1.7, *F*-change = 6.75, *p* = 0.02]. This means that more frequent Alternational code-switchers performed worse at inhibition in the flanker task condition interspersed with Insertions of German into English. This suggests that Insertion of German into English draws upon different processes than Alternation.

##### Predictors of performance in the dense code-switching mode

The Conflict effect measured in RTs was predicted by the independent variable IQ explaining 14.2% of performance variance [*R*(1,27) = 0.38, *R square* = 0.14, adj. *R square* = 0.11, *B* = −0.62, β = −0.38, *Constant* = 122.76, *F*-change = 4.46, *p* < 0.0001]. None of the linguistic variables predicted performance at the Conflict effect. When assessing the Conflict effect measured in Accuracy rates, Balance was a negative predictor of the Conflict effect, explaining 16.7% of performance variance [*R*(1,26) = 0.41, *R square* = 0.17, adj. *R square* = 0.13, *B* = 0.005, β = 0.41, *Constant* = 0.00, *F*-change = 5.19, *p* = 0.03]. This means that more balanced bilinguals produced less errors in the Dense code-switching condition. It can therefore be said that the more balanced bilinguals were, the better they performed at inhibitory control in the Dense code-switching condition. This makes sense given that balanced bilingualism tends to go hand in hand with more dense forms of code-switching (Muysken, 2000). It can thus be assumed that balanced bilinguals frequently train the proactive control modes engaged by Dense code-switching, explaining the positive correlation between Balance and inhibition in the Dense code-switching mode.

##### Predictors of performance in the bilingual modes overall

**FIGURE 7:**
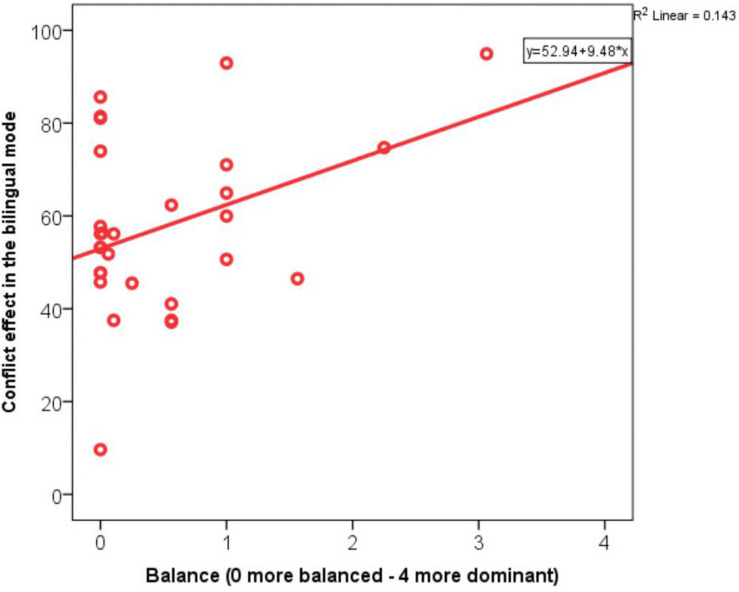
Correlation between language balance and the Conflict effect in the bilingual mode.

As illustrated by Figure 7, the regression analyses with the Conflict effect composite score (RTs) across all bilingual modes revealed that the only significant predictor of inhibitory performance was dominance [*R*(1,26) = 0.38, *R square* = 0.15, adj. *R square* = 0.11, *B* = 8.34, β = 0.38, *Constant* = 54.74, *F*-change = 4.61, *p* = 0.04]. Dominance was a negative predictor of inhibitory performance, meaning that more L1-dominant bilinguals performed less well at inhibition across the bilingual modes. The flipside of this is, that bilinguals that were more balanced performed better at inhibition across the bilingual modes. This is in line with the fact that the balanced bilinguals in Wu and Thierry’s study (2013) performed better in the bilingual mode.

##### Summary of regression analyses in the different code-switching conditions

A number of observations regarding the linguistic variables under study can be made to obtain a better understanding of the EFs involved in code-switching. Firstly, a negative correlation between Dense code-switching frequency and inhibitory performance at task blocks inducing Single-language and Alternational control modes was attested. Both of these control modes could be hypothesized to involve global and reactive control modes and macro-management of languages, whilst Dense code-switching may involve more local proactive control modes, hence the negative correlation. Secondly, there was a negative correlation between the frequency of Alternational code-switching and inhibitory performance in the two blocks designed to induce Insertional code-switching modes, suggesting that Insertion and Alternation differ in terms of the control modes involved. Crucially, dominance was a negative predictor of inhibitory performance in the bilingual mode conditions, in that more balanced bilinguals performed better in the control modes triggered by bilingual modes.

## Discussion

This study explored the impact of experimentally induced language modes (single-language and code-switching modes) on bilinguals’ EFs, and how these effects interact with more permanently entrenched EF modulations resulting from bilinguals’ regular code-switching habits. Participants were 29 German (L1)–English (L2) sequential bilinguals whose regular code-switching habits had been assessed using a frequency judgment task. Executive performance was tested in a flanker task inducing the following language modes by interspersing the flankers with sentences: (1) Single-language (L2 English), (2) Alternational code-switching, (3) Insertional code-switching English into German, (4) Insertional code-switching German into English, (6) Dense code-switching. In the single-language condition, bilinguals’ EF performance was compared to that of 29 monolingual participants. It was predicted that the control modes activated by the different language modes would transfer to performance at the non-verbal flanker task (Wu and Thierry, 2013). The study also investigated the effects of language dominance on EF performance in the different language modes. The following paragraphs will discuss the results in relation to each research question presented in the introduction. It should be noted that our sample size was small, so any conclusions drawn from our findings must be interpreted with caution (Paap, 2014).

Our first hypothesis was that our L1-dominant bilinguals would display enhanced inhibitory performance in the L2-single-language condition due to heightened levels of inhibition required to suppress the L1. We found converging evidence for this prediction from both Accuracy and RT comparisons. Bilinguals performed better in the single-language, compared to the four bilingual conditions with respect to overall RTs and Accuracy in the incongruent trials. Accuracy rates in the single-language mode were similar for incongruent and congruent trials, i.e., participants experienced no conflict effect at all. Moreover, they performed better in the code-switching mode involving the suppression of the dominant L1, i.e., “Insertion German into English” using the L2 English as the matrix language, than in the other code-switching conditions. This finding is in line with the notion that sequential bilinguals activate inhibitory schemata to suppress their L1 in L2 monolingual contexts (Meuter and Allport, 1999; Costa and Santesteban, 2004; Filippi et al., 2014), and that suppressing the L1 is more effortful than suppressing the L2 (see Bobb and Wodniecka, 2013, for discussion). It is also in line with processing models of bilingual language production, such as the ACH and the CPM, which suggest that code-switching recruits less inhibition and different control modes than single-language modes (Treffers-Daller, 2009; Green and Abutalebi, 2013; Green and Wei, 2014). Moreover, bilinguals’ better performance at overall RTs in the single-language mode suggests that the single-language mode boosts not only inhibition, but also monitoring. This is plausible, given that proactive inhibitory processes go hand in hand with matching proactive monitoring processes (Botvinick et al., 2001).

The second prediction of this study was that the L1-dominant bilinguals would display evidence of greater inhibitory activation than monolinguals in the single-language version of the flanker task due to having to suppress their L1, whilst the monolinguals have no need to activate inhibition to suppress another language. This was indeed the case for Accuracy rates. Monolinguals displayed a greater conflict effect than bilinguals, indicating less strong inhibitory activation. There was also a slight tendency for bilinguals to outperform monolinguals on Accuracy in incongruent trials, but this was only a marginal trend. It has been argued that it is the inhibitory effort expedited to suppress non-target languages in single-language modes which trains EFs in bilinguals, and ultimately leads to performance differences between bilinguals and monolinguals (Bialystok, 2009). The results from our study support this notion. It is also possible that permanently entrenched effects of bilingualism contributed to the observed performance differences. The bilingual “advantage” in a verbal task condition is at odds with previous reports of bilingual disadvantages in verbal tasks (Bialystok, 2009; Kharkhurin, 2010) and supports recent reports that bilinguals also have linguistic advantages in verbal tasks challenging inhibition (Teubner-Rhodes et al., 2016). Interestingly, performance differences between bilinguals and monolinguals occurred in Accuracy, not in RTs. This is in line with the Wu and Thierry (2013) study, which found language mode to affect Accuracy, but not RTs. However, this finding needs to be considered with care because the Accuracy rates were close to ceiling, so any observed effects were very small. When drawing conclusions about the comparison between monolinguals and bilinguals, one also needs to bear in mind that the administration order of the flanker tasks was slightly different for the two groups (cf. section “Assessing the Dependent Variable Executive Control”).

Our third research question explored the fast-modulation effects of code-switching modes on EF performance, predicting that bilingual modes involving reactive control (Alternation, Insertion) should lead to better inhibitory performance (Conflict effect), whilst bilingual modes activating proactive control modes should lead to better monitoring performance (overall RTs). However, when comparing EF performance across the flanker conditions inducing different types of code-switching modes, no statistically significant effects were observed for either inhibitory control (Conflict effect) or for monitoring (overall RTs). Nevertheless, several interesting observations were made about trends. Firstly, overall RTs measuring proactive monitoring and the conflict effect measuring inhibitory performance followed the same, not opposite, patterns across the four blocks. Therefore, there did not seem to be a dissociation between the two aspects of EFs in terms of overall performance. This suggests that inhibitory and monitoring processes are intricately related (Costa et al., 2009). Secondly, bilinguals performed best in conditions inducing language modes using the L2 as the matrix language, supporting the notion that the inhibition of the dominant L1 is effortful and boosts EF performance. Thirdly, the finding that conditions using the L2 English as the only language (single-language condition) or as the main matrix language (Insertion from German into English) had the lowest RTs suggests that this experiment might be tapping into something more global: conditions where the L1 is most strongly inhibited (presumably involving greater inhibitory control effort) may be attenuating the cost of resolving subsequent conflict in incongruent conditions. Moving forward, it would be informative to examine this interaction with L1-monolingual and L2-monolingual blocks as well as a mixed block with “inter-sentential” switches inducing a dual control mode in the sense of the ACH. In this study, we did not have a condition inducing a dual language mode, so no conclusions can be drawn about the predictions of the ACH regarding dual language contexts and no direct comparison can be made to studies that investigated dual language contexts (Sanchez-Azanza et al., 2020).

The fourth research question related to the interaction of permanently entrenched EF modulations through regular code-switching practices with experimentally induced language modes. To investigate this, we conducted multiple regressions with inhibitory performance in the different language modes as outcome variables and regular code-switching habits as predictor variables. We also investigated the impact of general cognitive abilities and language background variables as predictors. The different language mode conditions differed in terms of the variables explaining inhibitory performance. In line with predictions, there was a negative correlation between bilinguals’ frequency of Dense code-switching and inhibitory performance in task blocks inducing non-corresponding reactive control modes, i.e., Alternational control modes. This means that the more frequently bilinguals engaged in Dense code-switching, the less well they performed at task conditions associated with reactive control modes. This suggests that fundamentally different control processes are involved in Dense code-switching (proactive control modes) compared to Alternation (reactive control modes). This observation is in line with previous studies investigating intra-sentential code-switching and EFs, which suggested that qualitatively different control modes are involved in different types of code-switching (Hofweber et al., 2016, 2019, 2020). Moreover, there was a negative correlation between the frequency of Alternational code-switching and inhibitory performance in the two conditions inducing Insertional code-switching modes, calling into question a grouping of Alternation and Insertion into a common coupled control mode category (Green and Wei, 2014).

In view of these findings it is clear that further tests of the assumptions of existing processing models of code-switching (Green and Abutalebi, 2013; Green and Wei, 2014) are needed. According to the CPM model of code-switching and the ACH, Dense forms of code-switching are neutral with respect to most of the control processes involved in speech production, except for opportunistic planning, by which they mean “making use of whatever comes most readily to hand in order to achieve a goal” (Green and Abutalebi, 2013, p. 519). As the informants in the current study did not engage in Dense code-switching that frequently it is difficult to test this hypothesis on the basis of the current evidence. Further research particularly from communities where Dense code-switching is widely practiced is needed to shed new light on the relationship between cognitive control and Dense code-switching.

To investigate the impact of language dominance on bilinguals’ inhibitory performance, bilinguals’ dominance was entered as a predictor into the regression. Aside from regular code-switching frequency scores, the most prominent other predictor variable explaining inhibitory performance variance was bilinguals’ language dominance. L1-dominance correlated negatively with inhibitory performance in all bilingual conditions inducing code-switching modes, apart from the Alternational mode block. The more L1-dominant our overall L1-dominant bilinguals were, the less well they performed in the bilingual task conditions. In other words, more balanced bilinguals performed better at inhibition in the code-switching conditions. The influence of the dominance variable underlines the importance of this factor in modulating EFs (Treffers-Daller, 2016). A possible explanation is that balanced bilinguals engaged more frequently and more Densely in code-switching (Muysken, 2000), so they practiced the proactive forms of control activated in code-switching modes. A plethora of linguistic studies devoted to codeswitching have in fact noted that bilinguals who are highly proficient in both languages typically favor complex intra-sentential codeswitches and exhibit greater consistency of codeswitching occurrences, whilst less proficient bilinguals tend to limit switching to freely movable constituents (e.g., tag items; Poplack, 1980) and show less voluntary control of their switching behavior (Lipski, 2014).

When comparing the results from this study to those of other studies using similar experimental paradigms (Wu and Thierry, 2013; Jiao et al., 2019; Adler et al., 2020), the most salient difference is that we observed a reversed pattern of relative performance in bilingual and single-language modes. Whilst the bilinguals in previous studies performed better in the bilingual than in the monolingual flanker task conditions, the bilinguals in our study displayed better inhibitory performance in the single-language mode. This discrepancy may either be due to differences in the nature of the experimental design or in bilinguals’ sociolinguistic backgrounds. In both the Wu and Thierry (2013) and the Jiao et al. (2020) studies, stimuli in the mixed language block alternated between languages, which arguably induced an alternational mode associated with reactive control modes. The Adler et al. (2020) study differed from ours in that they administered single language and bilingual stimuli within the same block, whilst we presented different code-switching types in a blocked design. These subtle experimental differences may account for differential outcomes. In addition, our bilinguals had a unique language dominance pattern which may explain their performance. The participants in the present study were sequential bilinguals who were dominant in their L1, whilst Wu and Thierry’s (2013) Welsh–English bilinguals were balanced bilinguals. Hence, they did not have a dominant L1 that required increased inhibitory effort for it to be suppressed in the monolingual context.

At the same time, it is important to note that in the present study, bilinguals’ performance in the code-switching conditions was modulated by dominance, i.e., the more balanced bilinguals amongst this L1-dominant group performed better in the bilingual mode conditions. The positive correlation between balance and EF performance in the bilingual conditions is in line with Wu and Thierry (2013) finding their balanced bilinguals to excel in the bilingual mode condition. This suggests that both balanced and dominant forms of bilingualism modulate EFs. However, they may impact different aspects of the executive system. Whilst balanced bilingualism enhances the more proactive forms of control required during code-switching, dominant bilingualism may enhance the more reactive, global and asymmetric forms of inhibition required to suppress a dominant L1 in monolingual contexts. This effect could further be strengthened by the fact that language dominance impacts code-switching patterns (Beatty-Martínez et al., 2020). Balanced bilingualism may enhance proactive forms of control because balanced bilinguals favor complex intra-sentential codeswitches and exhibit greater consistency of codeswitching occurrences, whilst unbalanced bilinguals tend to limit switching to freely movable constituents (Poplack, 1980), and show less voluntary control of their switching behavior (Lipski, 2014). Further insights into the relationship between language dominance and EFs could be gained by controlling for directionality of alternational code-switching, to assess whether switching into the L1 or into the L2 triggers greater inhibitory activation.

Immersion has been shown to modulate the relationship between bilingualism and EFs (Dussias and Sagarra, 2007; Linck et al., 2009; Baus et al., 2013; Beatty-Martínez et al., 2020). In this study, we attempted to tease apart the effects of different bilingualism variables on EFs by entering them as separate predictors in the regression. Our analyses isolated dominance and code-switching, but not immersion itself as a predictor of EF performance in the bilingual modes. Hence, it is possible that EFs are not shaped by immersion itself, but by its actual sociolinguistic consequences and linguistic correlates, such as shifts in language dominance or changes in code-switching patterns. In line with this reasoning, we observed that bilinguals’ language dominance patterns shifted as a result of immersion, so immersion had an indirect influence on EF performance, mediated by dominance patterns. Future research using immersion as a predictor variable should therefore consider breaking down the notion of immersion into its component parts and associated bilingualism variables to narrow down which precise aspect of the sociolinguistic consequences of immersion shape EFs. In this context, it may be interesting to contrast not only linguistic factors, but also cultural factors related to multilinguals’ degree of identification with their respective cultural backgrounds (Treffers-Daller, Ongun, Hofweber, and Korenar, this volume). The bilinguals examined in this study all share the experience of living in a context that favors the use of their L2 and restricts the use of their L1. Previous research highlights the complexity of the interplay between L1 down-regulation and L2 up-regulation during L2 immersion (e.g., Zirnstein et al., 2018). Future research should therefore consider how different patterns of association may emerge for other bilingual phenotypes (e.g., German–English codeswitching bilinguals immersed in their native language). In terms of non-linguistic predictor variables, it was interesting to note that the phonological working memory scores from the digit span administered in the English language came out as a significant predictor of inhibitory control in the task block inducing an English Matrix language mode. Bilinguals who displayed greater capacities at English-language Working memory therefore also displayed better inhibitory performance in the task condition using English as the matrix language of code-switching. The fact that working memory predicted inhibitory performance is in line with Engle’s (2002) model postulating that inhibition and working memory are interrelated components of EFs.

The interpretation of results in this study is complex because there is a multitude of factors interacting. Moreover, several study limitations need to be addressed by future research. Firstly, we observed a lack of clear fast-modulation effects for the different code-switching modes. This could have been due to the stimuli having been administered only in the visual format, when code-switching is more typical of spoken registers. Code-switches in the stimuli were also highlighted by bold font, which may have heightened bilinguals’ consciousness of the code-switches. Future research could thus increase the ecological validity and effectiveness of the stimuli by presenting them in an aural format, as was done by Hofweber et al. (2019). An alternative explanation for the absence of a clear effect of code-switching mode on EFs is that in reality different code-switching types and monolingual sentences co-occur within the same conversation. Therefore, a blocked design represents an abstraction from bilinguals’ sociolinguistic reality. Future research could address this by adopting a design in which code-switches and monolingual stimuli are displayed in an alternating fashion, following Adler et al. (2020). Moreover, subtle EF fast-modulations may have been left undetected by our behavioral experiment. Future research on intra-sentential code-switching and EFs could thus use tools that are more sensitive to the cognitive processes underlying performance, such as EEG. Finally, the sample size of this study was small, so a lack of power may have influenced results. A small sample size may not only reduce the power to detect significant findings, but it may also increase the probability of spurious findings (Paap, 2014). It is therefore essential to conduct further research investigating the interaction between code-switching and EFs in larger bilingual populations.

In terms of the task sensitivity of the flanker task, it is important to note that performance differences in this paradigm occurred predominantly in Accuracy (not RTs) both in this study and in Wu and Thierry’s (2013) study. However, Accuracy in the flanker was very high in this study. The near-ceiling effect in Accuracy means that any observations based on Accuracy need to be interpreted with caution as they represent very small differences. It also reduces the probability of observing reliable differential effects by condition. Future studies could thus investigate the reliability of the observed effects in Accuracy by increasing the difficulty of the flanker task, e.g., by reducing the stimulus presentation duration or the time frame given for responses. This may lead to greater variability in error numbers, which may lead to stronger Accuracy-based results. Moreover, to reduce the duration of the experimental protocol, we only administered a flanker task with a 50–50 congruent-incongruent trial split, inducing a proactive control mode. To truly tease apart reactive and proactive control modes, future research should manipulate the trial split of the flanker task, as was done by Hofweber et al. (2020). A further limitation of this study is that it used only a flanker task to assess EFs. This means that we only tapped into the inhibitory sub-component of EFs. To adequately take into consideration the complexity of EFs, future research should investigate fast-modulation effects on shifting and task-switching, which are crucial aspects of EFs (Miyake et al., 2000).

Another interesting avenue for further research is to investigate the relationship between the social diversity of language use and EF performance under different experimentally induced language modes. Our study focused on how different code-switching types map onto EF performance. However, it would also be interesting to explore how the variety of code-switching strategies used in bilinguals’ everyday life influences EF performance under different language mode conditions. The social diversity of language use within the community has been shown to influence EFs (Gullifer et al., 2018). A study by Beatty-Martínez et al. (2020) observed that bilinguals’ cognitive control engagement strategies ranged across the proactive-reactive continuum with bilinguals who kept their languages separate exhibiting a greater reliance on reactive control and bilinguals living in a more variable environment (with respect to the types of conversational exchanges) showing a greater reliance on contextual information, favoring an engagement of proactive control. Likewise, it would be interesting to investigate the impact of language entropy, using new measures designed to capture differences in bilinguals’ social experience in such a manner (Gullifer and Titone, 2020a).

Finally, the sequential bilinguals in this study performed better in the single-language mode than in the bilingual modes. This better performance pertained when they were compared to a monolingual group. This suggests that temporary fast modulation effects through different language mode requirements can ultimately result in more long-lasting neural plasticity effects re-shaping executive functioning. However, it is also possible that the bilingual advantage in the single-language mode was a temporary effect due to having to suppress the L1. To fully answer the question whether fast-modulation effects translate into permanently entrenched effects, the bilinguals would need to be compared to L1 monolinguals in a verbal flanker tasks using L1 stimuli or in a non-verbal flanker task.

## Conclusion

This study focused on two aspects that have repeatedly been put forward as sources of variability in bilinguals’ EF performance: (1) the interactional context or language mode in which bilinguals operate and (2) bilinguals’ language dominance profiles. We assessed the impact of different language modes on bilinguals’ EFs by inducing different language modes (single-language mode; different code-switching modes) in a flanker task measuring inhibition. EF performance in the different language modes was then related to bilinguals’ regular code-switching habits and their language dominance profiles. Our L1-dominant bilinguals performed better in the L2-single-language compared to the bilingual conditions as they activated inhibitory schemata to suppress their L1. This EF modulation also translated into performance differences when comparing the bilinguals to a monolingual control group, suggesting that bilinguals draw upon inhibition when managing linguistic co-activation. Whilst EF performance in the single-language and bilingual modes differed significantly, there was no significant difference in EF performance across the different code-switching modes. The task conditions inducing different code-switching modes differed only in terms of the bilingualism-related variables predicting inhibitory performance, notably regular code-switching habits and dominance. Frequency of Dense code-switching was a negative predictor of performance in the condition activating Alternational and Monolingual control modes. This suggests that Dense code-switching may involve (proactive) control modes that are different from those activated in Alternation and Single-language modes (reactive control modes). Importantly, bilinguals’ language dominance played an important role in explaining EF performance patterns. The less L1-dominant and therefore more balanced bilinguals displayed better inhibitory performance in the bilingual conditions. This highlights the importance of assessing both language usage and dominance patterns in bilingualism research and underlines the complexity of the interactions that need to be considered when researching bilingualism and EFs.

## Data Availability Statement

The raw data supporting the conclusions of this article will be made available by the authors, without undue reservation, to any qualified researcher.

## Ethics Statement

This study was reviewed by the University of Reading School of Psychology and Clinical Language Sciences’ Research Ethics Committee and was given a favorable ethical opinion for conduct (2014-105-TM).

## Author Contributions

JH: first author. TM and JT-D: project supervisors. All authors contributed to the article and approved the submitted version.

## Conflict of Interest

The authors declare that the research was conducted in the absence of any commercial or financial relationships that could be construed as a potential conflict of interest.

## Footnotes

1 The notion of entropy is similar to that of Grosjean’s (2016) Complementarity Index, but computed in a slightly different way, and across fewer domains than Grosjean’s CI.
